# Discrete Subaortic Stenosis: Perspective Roadmap to a Complex Disease

**DOI:** 10.3389/fcvm.2018.00122

**Published:** 2018-09-13

**Authors:** Danielle D. Massé, Jason A. Shar, Kathleen N. Brown, Sundeep G. Keswani, K. Jane Grande-Allen, Philippe Sucosky

**Affiliations:** ^1^Department of Mechanical and Materials Engineering, Wright State University, Dayton, OH, United States; ^2^Department of Bioengineering, Rice University, Houston, TX, United States; ^3^Division of Pediatric Surgery, Texas Children's Hospital, Houston, TX, United States; ^4^Department of Surgery, Baylor College of Medicine, Houston, TX, United States

**Keywords:** discrete subaortic stenosis, congenital heart disease, hemodynamics, etiology, left ventricular outflow tract, wall shear stress, aortoseptal angle

## Abstract

Discrete subaortic stenosis (DSS) is a congenital heart disease that results in the formation of a fibro-membranous tissue, causing an increased pressure gradient in the left ventricular outflow tract (LVOT). While surgical resection of the membrane has shown some success in eliminating the obstruction, it poses significant risks associated with anesthesia, sternotomy, and heart bypass, and it remains associated with a high rate of recurrence. Although a genetic etiology had been initially proposed, the association between DSS and left ventricle (LV) geometrical abnormalities has provided more support to a hemodynamic etiology by which congenital or post-surgical LVOT geometric derangements could generate abnormal shear forces on the septal wall, triggering in turn a fibrotic response. Validating this hypothetical etiology and understanding the mechanobiological processes by which altered shear forces induce fibrosis in the LVOT are major knowledge gaps. This perspective paper describes the current state of knowledge of DSS, articulates the research needs to yield mechanistic insights into a significant pathologic process that is poorly understood, and proposes several strategies aimed at elucidating the potential mechanobiological synergies responsible for DSS pathogenesis. The proposed roadmap has the potential to improve DSS management by identifying early targets for prevention of the fibrotic lesion, and may also prove beneficial in other fibrotic cardiovascular diseases associated with altered flow.

## Clinical presentation

**Figure 1:**
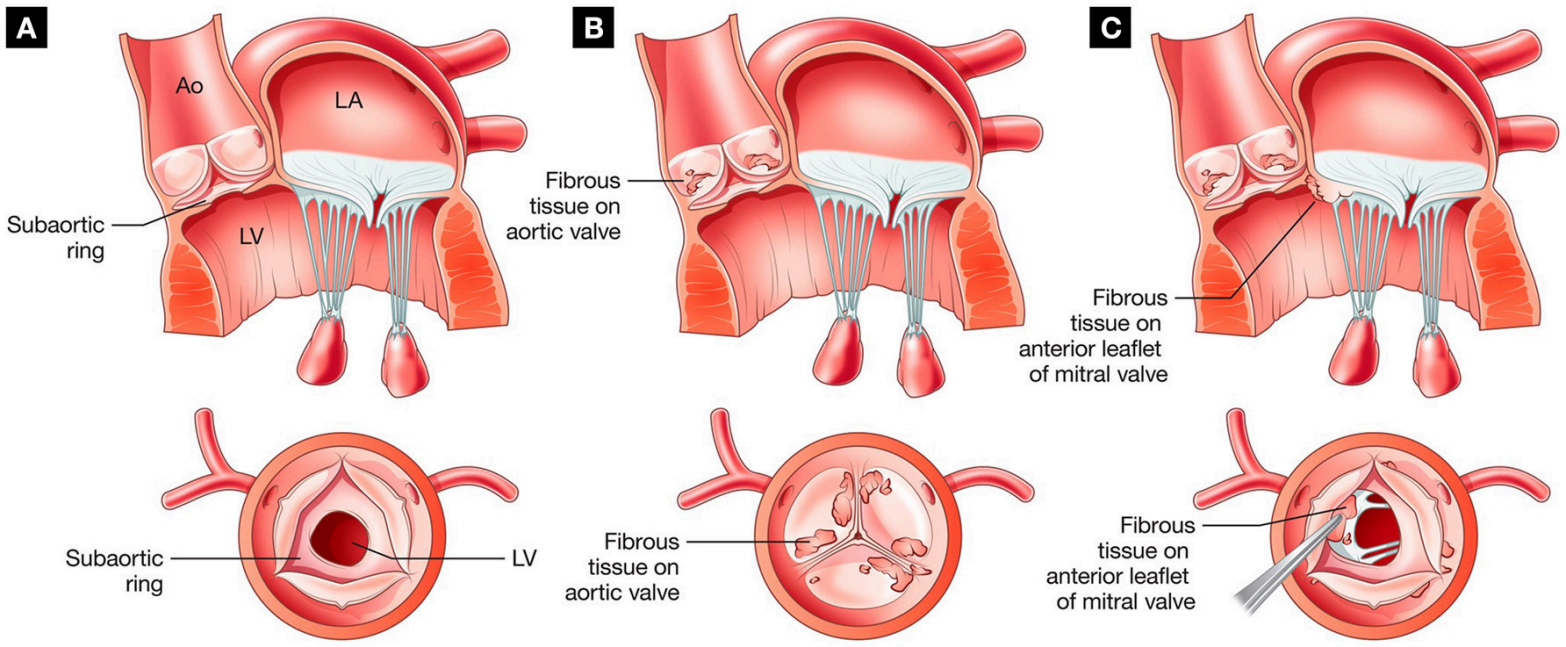
Different presentations of DSS showing: **(A)** isolated geometry; **(B)** involvement with the aortic valve; and **(C)** involvement with the mitral valve (Ao, aorta; LA, left atrium; LV, left ventricle). Adapted from (4) with permission from Elsevier.

Discrete subaortic stenosis (DSS) is a congenital heart disease characterized by the formation of a fibrous membrane obstructing the left ventricular outflow tract (LVOT). DSS occurs within about 6% of children with congenital heart defects (1, 2) and is responsible for 8–30% of total LVOT obstructions in children and up to 20% of obstructions that require intervention (3, 4). Key features of the disease are its rapid progression and its association with both a high-velocity jet and a high-pressure gradient across the LVOT (5–8). The membrane that causes DSS can present with a variety of morphologies. It is most commonly described as a fibromuscular ring of tissue, but can also present as an incomplete shelf or ridge-like structure (5, 9, 10). The lesion consists of five tissue layers: (1) endothelial layer, (2) glycosaminoglycans in the sub-endothelial layer, (3) fibroelastic

layer with collagen bundles and elastin fibrils, (4) smooth muscle layer with a thickened basement membrane, and (5) fibrous layer with increased collagen (11). The location of this membrane can range from just below the aortic valve where it sometimes fuses with the leaflets, to lower within the LVOT where it can become attached to the anterior mitral valve leaflet (Figure 1) (12). Without intervention, DSS can result in left ventricle (LV) hypertrophy and dysfunction, aortic regurgitation (AR), endocarditis, arrhythmias, and death (2, 5, 13–16).

DSS develops typically within the first decade of life and little is understood about its early development (17). The disease can present concomitantly with a variety of other congenital cardiac defects, such as bicuspid aortic valve (23% of DSS patients), ventricular septal defect (37%), and Shone's complex (4, 8, 15). Early diagnosis can be difficult as most patients remain asymptomatic throughout the disease (10, 17, 18). When present, symptoms include chest pain, heart failure, and/or syncope (8, 18). Nevertheless, DSS is usually revealed by the existence of both a mid-systolic and an end-diastolic murmur during physical examination. Diagnosis is given during a follow-up 2D Doppler echocardiography examination of the flow within the LVOT. However, cases have been reported of patients with no significant echocardiographic LVOT abnormalities in early childhood who later developed DSS at a relatively rapid rate (12, 19).

Risk factors promoting initial occurrence include morphological LVOT abnormalities such as sharp aortoseptal angle (AoSA), subphysiologic aortic annulus diameter, and large aortic valve-mitral valve separation distance (12, 16, 20, 21). Recurrence, which occurs in 8–34% of patients over a 10 year period (12, 22–27), has been associated with young age at both initial diagnosis and surgical intervention, smaller aortic annulus, proximity of the obstruction to the aortic valve, and a higher preoperative peak LVOT gradient (4, 14, 16, 25). Females have a 1.5 times greater risk of recurrence compared to males (8, 16). This increased risk could be associated with the smaller LVOT anatomies in women, but the role of gender in DSS recurrence has not been investigated (16).

## Disease management

Surgical resection of the membrane alleviates the obstruction but is associated with a high rate of recurrence (12, 22–27), which has fueled the debate on timing of intervention. While some studies advocate for immediate resection to help prevent later progression and complications, others argue that resection should be considered only under certain conditions (2, 8, 12, 28). The general consensus reported in the 2008 ACC/AHA guidelines recommends surgical intervention for peak instantaneous echocardiographic gradient greater than 50 mmHg, mean gradient greater than 30 mmHg, or catheter measurement of the resting peak-to-peak gradient greater than 50 mmHg (29). Support for early intervention stems from the demonstrated success of surgery in preventing AR, especially in infants (9, 22, 24, 25, 30), while that for delayed intervention cites the potential for increased AR severity, mitral valve damage and heart block as major risks of early resection (5, 28, 30–32). Specific resection techniques range from simple removal of the membrane to a more aggressive approach combining membrane resection and myectomy (12, 17, 33). Although both effectively provide relief, the more aggressive approach has been associated with a lower instance of recurrence (25, 32) but a higher risk for iatrogenic ventricular septal defect and heart block (4, 9, 24, 26). Regardless of the surgical technique, mortality during membrane resection is reported to be approximately 3% (16, 22, 24, 34, 35).

## Emergence of a hemodynamic etiology

### Description

The mechanisms behind DSS pathogenesis have been subject to debate since the disease was first discovered. Initially, DSS was classified as a congenital disorder. While there are still claims that Mendelian autosomal recessive inheritance is the main mechanism behind DSS, such occurrence is rare (12, 36–38). The lack of a clear inheritance pattern along with the established association of DSS with LV morphological abnormalities has classified DSS as an acquired obstruction resulting from adverse LVOT flow patterns (12, 20, 39). This hemodynamic etiology, which hypothesizes the existence of mechanobiological synergies between the septal wall and the surrounding fluid mechanical stresses, describes DSS pathogenesis as a three-step process: (1) existent or surgically-induced LV morphological abnormalities cause (2) LV flow derangements and mechanical stress overloads on the septal wall, which trigger in turn (3) a fibrotic cellular response leading to membrane formation (9, 12, 30, 40). While this theory clearly identifies hemodynamic factors as drivers of DSS pathogenesis, it does not preclude the possible contribution of genetics in promoting a fibrotic response.

### Supporting evidence

Clinical studies and case reports have used transthoracic and transesophageal echocardiography to characterize deranged flow patterns, supporting a role for hemodynamics in the pathogenesis of DSS (4, 14, 16, 25, 41, 42). Preoperative flow characteristics in DSS patients include high mean and peak LVOT gradients, marked subaortic acceleration, and transition to turbulence (42, 43). While resection results in a decrease in LVOT pressure gradient (4, 14, 16, 25), long-term follow-up assessments have evidenced a progressive linear increase in peak LVOT gradient over time (4). Regardless of its cause, an increased LVOT gradient will be associated with flow acceleration, which will translate into an increase in the interfacial force or wall shear stress (WSS) experienced by the LVOT and the subaortic region of the septal wall. These observations suggest the existence of underlying LV hemodynamic abnormalities, even in the absence of DSS lesion in those patients, which could contribute to both initial occurrence and recurrence.

While the impact of such WSS overloads on septal wall biology remains largely unknown, the knowledge gained from previous vascular mechanobiological studies offers some perspective. WSS plays a major role in cardiovascular disease through alteration of endothelial cell (EC) phenotype and loss of tissue homeostasis (44–46). *In vitro* studies on the effects of WSS on cardiovascular tissue and cells have demonstrated that vascular and valvular ECs interact with their surrounding mechanical environment to drive critical cell-extracellular matrix (ECM) processes. Studies have demonstrated the ability of low WSS to increase EC migration, permeability, proliferation, and activation of certain transcription factors. Regions of accelerating flow with high WSS and positive WSS spatial gradients promote ECM degradation and cellular loss, while arterial regions experiencing high WSS but negative WSS spatial gradients are protected from ECM degradation (47, 48). Those studies provide evidence for the distinct sensitivity of the vascular endothelium to WSS magnitude and spatial gradient, and the vulnerability of vascular endothelial regions subjected to elevated WSS and positive spatial gradients to inflammation, remodeling, and matrix degeneration. The sensitivity of ECs to mechanical forces combined with the histological hallmarks of DSS lesions, which include inflammation, fibrosis, and myofibroblast proliferation (12, 49, 50), suggests the activation of similar biological cascades in response to the LV flow derangements typically observed in DSS patients. More specifically, ECs subjected to WSS respond by triggering molecular pathways involving reactive oxygen species, nitric oxide, miRNAs, and growth factors (51–53). Activation of mechanosensing receptors in ECs, EC-smooth muscle cell (SMC) crosstalk, and signaling pathways in response to laminar vs. disturbed flow are rigorously investigated areas in vascular biology (52, 54–56). These processes are often mediated by PECAM-1, an EC adhesion molecule (52, 57). Vascular ECs under WSS communicate with underlying SMCs, which contribute to remodeling of the surrounding tissue and the ECM (51, 52, 54, 56). Disturbed flow is known to alter EC-SMC communication and behavior in blood vessels, but most factors in this process have yet to be characterized for endocardial ECs and their milieu (52, 55, 58).

## Knowledge gap and research needs

Although the hemodynamic etiology of DSS is now relatively well accepted, its rigorous validation is still lacking. In the nearly 400 publications on DSS to date, most have focused on the echocardiographic description of LV/LVOT hemodynamics, the investigation of genetic inheritance, or the clinical and biochemical descriptions of DSS lesions. The realization that DSS pathogenesis may stem from a combination of hemodynamic and genetic cues motivates the implementation of higher-level approaches. Future research should focus on the detailed characterization of hemodynamic stresses associated with steepened AoSAs, and the elucidation of the underlying mechanobiological cellular and molecular mechanisms of DSS. Similar approaches have been successful at shedding light on the pathogenesis of calcific aortic valve disease (59–62), atherosclerosis (52, 63, 64), and bicuspid aortic valve aortopathy (65–67). The following sections present some perspective on the potential implementation of similar strategies in the context of DSS.

### WSS characterization in abnormal LV anatomies

Echocardiography has evidenced the existence of global LV flow alterations in DSS patients pre- and post-resection (4, 14, 16, 25, 41, 42). Although this modality provides sufficient information for diagnostics and retrospective analysis, it is unable to capture the local flow characteristics due to its limited temporal and spatial resolutions. The elucidation of the potential synergies between the endocardial endothelium and the flow alterations associated with abnormal LV morphologies requires the implementation of more resolved approaches capable of capturing the WSS imposed by the surrounding blood flow on the septal wall. Computational and experimental techniques have been developed to quantify WSS in other cardiovascular disorders and could be implemented in the context of DSS.

Computational fluid dynamics (CFD) is a technique capable of predicting the flow characteristics at high spatial and temporal resolutions by modeling mathematically the governing equations of fluid motion. This approach consists of the creation of a three-dimensional geometry reconstructed from clinical patient images, the prescription of boundary conditions at the inlets and outlets of the model, and the numerical solution of the linearized flow equations (68). The availability of increasingly powerful workstations has enabled fluid-structure interaction (FSI) simulations that not only solve for the flow but also account for the deformation of surrounding structures and its impact on the flow (69). Such an approach is well adapted to the characterization of pulsatile flow in compliant cardiovascular structures and has been successfully implemented to capture WSS characteristics in ventricular (70–75), valvular (76–78), and aortic flows (44, 79).

**Figure 2:**
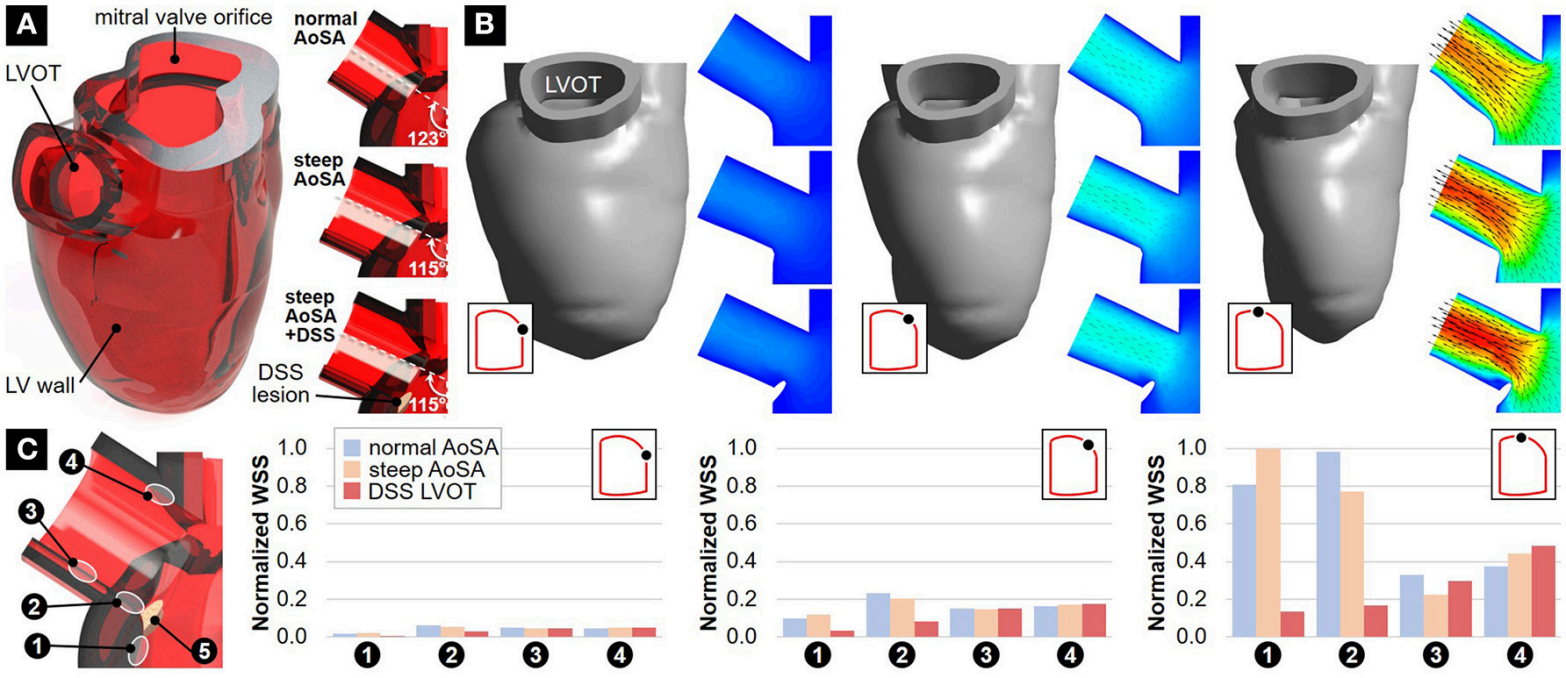
Preliminary FSI modeling in normal and DSS LVs: **(A)** geometrical models with normal AoSA, steepened AoSA and DSS lesion; **(B)** velocity predictions at early, mid-peak and peak ejection (inset: LV pressure-volume curve); and **(C)** WSS predictions at early, mid-peak and peak ejection.

Our group has recently evidenced the applicability of this computational strategy to the characterization of the hemodynamic impact of a steep AoSA in a contracting human LV reconstructed from multiple-slice cine magnetic resonance images (80). Three cases were considered: (1) normal AoSA, (2) steepened AoSA, and (3) steepened AoSA with a DSS membrane. The obstruction was represented as a thin semi-lunar membrane attached at the base of the LVOT (Figure 2A). Two-way coupling between the deforming LV and the flow was achieved in ANSYS 18 (ANSYS Inc). LV wall mechanics were modeled using an isotropic linear elastic formulation with mechanical properties representative of the average passive/active properties obtained from the literature (81, 82). LV ejection was simulated by imposing a time-dependent pressure condition on the LV wall and a wall condition on the mitral valve orifice. While the steepened AoSA generated the same LVOT flow structure as the normal AoSA, it resulted in a 6% increase in LVOT velocity at peak ejection (Figure 2B). The presence of the DSS lesion generated substantial flow alterations characterized by a recirculation bubble immediately downstream of the lesion, increased LVOT jet skewness toward the lower wall, and stenotic conditions marked by a 12% increase in maximum LVOT velocity at peak ejection. In addition, the steepened AoSA resulted in WSS abnormalities both upstream and downstream of the site prone to DSS lesion formation (24% increase in region 1, 22% decrease in region 2, vs. normal LV) (Figure 2C). These preliminary models illustrate the feasibility and benefits of CFD for the hemodynamic characterization of DSS, and suggest the existence of supra-physiologic WSS levels on the endocardial region prone to DSS lesion formation.

Experimental flow techniques have also been implemented to measure LV hemodynamics *in vitro*. Particle image velocimetry (PIV) is a laser technique employing optical and statistical methods to measure the instantaneous velocity field in a section of the flow by capturing the average displacement of tracer particles captured in two successive flow images (83). This technique has been adapted to the characterization of the pulsatile and turbulent flow characteristics in heart valves (84–86), the aorta (87–89), and the LV (90–92). These studies typically consist of a realistic and compliant silicone model of the structure of interest connected to a flow loop generating physiologic pressure and cardiac output. PIV measurements in the LV have been performed to capture the temporal flow velocity, vorticity dynamics, and turbulence characteristics in a deforming LV (90). Although the limitations inherent to PIV prevent its use toward the capture of near-wall flow characteristics such as the regional endocardial WSS, PIV is well adapted to the assessment of bulk flow characteristics. Therefore, similar setups could be envisioned for the measurement of turbulent flow in patient-specific DSS LVs and LVOTs, which will be needed for the validation of the computational results.

### Elucidation of septal wall mechanobiology

While the existence of flow derangements and WSS abnormalities in LV morphologies prone to DSS development has been partially established *in vivo*, their ability to trigger a fibrotic response in the endocardium also needs to be rigorously investigated. Diverse benchtop approaches allow for the application of WSS to cells, yet a major obstacle is the challenge to replicate *in vitro* the full spectrum of the native flow characteristics, which include three-dimensionality, pulsatility, and multidirectionality. Previous investigations of the biological response of ECs to WSS have implemented cone-and-plate bioreactors. These devices consist of an inverted cone rotating above a stationary plate supporting the cells (93). The rotation of the cone generates a flow of culture medium above the plate, resulting in the generation of WSS on the cell monolayer, making it a convenient tool for the production of a well-controlled, pulsatile/oscillatory WSS environment. The need to elucidate the role of cell-ECM communication in WSS signaling has motivated the design of more sophisticated devices capable of subjecting whole pieces of tissue to time-varying WSS (94–96). The implementation of similar devices to subject endocardial ECs or septal wall tissue to the local WSS abnormalities captured experimentally or computationally in LV morphologies prone to DSS would provide new insights into the potential transduction of altered WSS into inflammatory and fibrotic responses. In addition to the cellular response to WSS, the cellular signaling pathway and LV elastic modulus should be addressed in future studies of DSS. Resected DSS membranes show evidence of proliferation, fibrosis, and ECM remodeling (11), which provides compelling motivation to investigate the complex cell-cell communication that elicits membrane formation and fibrosis.

Complementary benchtop approaches can be employed to assess how cellular phenotype, adhesion, and migration is influenced by the stiffness of their environment (97–99). In fibrotic diseases like DSS, ECM stiffness is increased by fibrosis, which then influences cell behavior. ECs grown on stiff substrates show increased migration, proliferation, and adhesion compared with cells grown on soft substrates (99). Biomaterials can be used to mimic the elastic modulus of normal and pathological tissues so that cellular behavior in these environments can be studied (99, 100). Biomaterials can also be synthesized to present biochemical signals that influence cell behavior (99, 101). Taken together, strategies to fabricate materials in a highly customized manner (99–106) would be advantageous to use to replicate key aspects of the diverse cell communities, cell communication, and ECM microenvironments in the subaortic LVOT, such as paracrine signaling and substrate stiffness, to determine their roles in DSS.

### Development of new DSS models

The current *in vivo* model used to study DSS is a canine animal model (11, 107–111). This model has limitations in understanding how DSS forms and the underlying mechanism of DSS. Researchers have identified an inherited genetic link associated with DSS, as shown with Newfoundland dogs (107). However, this model has not been able to identify a specific gene correlated with DSS, or if the disease is acquired or congenital (107). The presentation of DSS in dogs varies from humans so it is unclear if a canine model of DSS could clarify the mechanism of the disease (108). Therefore, a disease model that elucidates the complex physiological conditions of DSS is needed.

### Development of a CFD-based surgical optimization framework

Computational engineering tools have been used as diagnosis and surgical planning tools to assess patient-specific hemodynamics in the context of Fontan and valve replacement procedures (112, 113). Similarly, CFD could be used to assess the potential benefits of resection in DSS patients. Should hemodynamics be identified as a key player in DSS lesion formation, CFD could also be implemented as a diagnosis or predictive tool for patients considered at risk for developing the disease. In both cases, the clinical implementation of CFD will require formal validation based on a combination of *in vivo* (e.g., PC-MRI, echocardiography) and *in vitro* (e.g., PIV) flow measurements.

## Conclusions

DSS is a complex disorder that remains largely unexplained and difficult to treat. The risks posed by surgery along with the unpredictability of recurrence following resection justify the need to understand the underlying mechanisms of DSS. Advances in clinical imaging techniques, computational and experimental fluid dynamics, and mechanobiology provide new opportunities to elucidate the pathogenesis of this disease. The approach outlined in this paper, which suggests the use of engineering tools and clinical information in tandem, has the potential to address the molecular and cellular mechanisms of DSS, elucidate its fundamental biology, and provide new predictive capabilities that may ultimately improve patient-specific diagnosis and management. The execution of this novel strategy will require a dual expertise in both engineering and medicine, with clinicians recruiting potential patients and analyzing medical data, and engineers providing mechanical data and designing tissue culture systems.

## Author contributions

DM, JS, and KB conducted the literature review and wrote the paper. JS performed and analyzed the flow simulations. PS, KG-A, and SK edited the paper and conceived the work.

### Conflict of interest statement

The authors declare that the research was conducted in the absence of any commercial or financial relationships that could be construed as a potential conflict of interest.
